# Hermaphrodism, from a Medico-Legal Point of View

**Published:** 1875-10

**Authors:** 


### Translations.

### HERMAPHRODISM,

### FROM A MEDICO-LEGAL POINT OF VIEW.

*Translated from the French of Basile Poppesco,*

By E. WARREN SAWYER, M.D.,

Lecturer on Obstetrics, Rush Medical College, Chicago.

(Continued from page 706.)

### III. NEUTER HERMAPHRODISM.

A third variety of hermaphrodism is that which is designated under the name of neuter hermaphrodism; it has been attempted to include in this variety two different categories: 1, the persons whose sex is not easily determined, well pronounced; 2, those persons in whom one observes the simultaneous existence of the organs of both sexes; this variety is also called the bisexual hermaphrodism.

The first variety does not exist; there are no beings absolutely neuter, not having one sexual attribute; and nearly all the cases reputed to be such, should be included in the apparent hermaphrodism of the male sex. Such is, at least, the opinion of the medical jurist, and it is certain that in consultations that which is demanded of him in law should be adopted.

Prof. Tardieu does not admit a single authenticated instance of bisexual hermaphrodism, with coexistence of all the essential and accessory organs of the male and female sex. The fact has been admitted, however, by such authors as Maret, Meckel, Dugès, Is. Geoffroy Saint-Hilaire, Dutrochet; but it is true that, at this epoch, they were limited to an anatomical examination of the organs. We will make known our observations, however, in which the histological examination having been made by competent men, the simultaneous existence of the ovaries and testicles was established beyond question.

Maret* reported an instance of the simultaneous existence of the organs of both sexes; on one side, said he, the labium contained a veritable testicle, with the cord of the spermatic vessels, the vas deferens, and a seminal vesicle full of spermatic fluid. The right labium enclosed a membranous pouch, in which descended, when the abdomen was compressed with the hand in the right iliac region, an ovoidal body which was recognized as a uterus, without any communication with the external parts, but having one Fallopian tube and one ovary. "Hubert," says this author, "though he had the essential organs of both sexes, was not able to fill the functions of either; in vain did the testicle elaborate the semen when an imperforation of the penis opposed its emission; a Fallopian tube embraced in vain a well formed ovary, when the uterus was enclosed in a pouch without opening."

This singular variety in which the individual is found on one side of the body constructed after the type of the male sex, and on the other after that of the female sex, has

* Maret Mém. de l'Acad. de Dijon: *Vesiculas seminales et ovarium habens.*

been described by Is. Geoffroy Saint-Hilaire,* under the name of hermaphrodism with excess in the number of parts, *hermaphrodism bisexual or lateral.* Verdier and Sou have met with an analogous instance, as have also Colombo and Petit; these cases will be rather of the type that should be known under the name of androgynia; but the most commonly then, the arrest of development, especially in the case where the sexual system is double, belongs sometimes to the sphere of the masculine organs, sometimes to the feminine organs, in virtue of the law discovered by Serres, which he designated under the name of balancing the organs.

But, we repeat it, these varieties of hermaphrodism are more than exceptional; and we have enlarged upon them because of the scientific standing of the men who have called attention to them. Without the aid of the microscope, it appears to us very difficult to distinguish between an ovary and a testicle, especially when concerned with organs that are atrophied, compressed, and have lost not only their normal relations but all their external features and their distinctive microscopical characteristics. Our aim has been only to represent the ideas and the opinions sustained and learnedly elaborated by those who have preceded us.

In closing, I will name one other variety, designated under the name of superadded hermaphrodism, by Dutrochet: in this case, the deepest organs are of one sex, the medium organs are of the opposite sex, and the external organs are the association of both sexes.

From what precedes, we are able to conclude, in a medico-legal point of view, that hermaphrodism presents itself to the observer under two principal forms; the frequent one is that in which an individual of the male sex offers an arrest of development, with some general and local features which approach the female sex; the other rare and exceptional, in which the person of the

* Is. Geoffroy Saint-Hilaire. Recherches anat. et phys. sur l'hermaphrodism chez l'homme et les animaux; rapport de Dutrochet.

female sex has, simultaneously, an excess of development in one organ and an arrest of development in some others, with a predominance of the masculine conformation more or less marked.

### EMBRYOLOGY AND PATHOLOGICAL PHYSIOLOGY.

The preceding study would certainly remain in a state of confusion and be vaguely remembered if, in reviewing the facts, we could not at first sight with clearness and precision refer all these infinite varieties to an arrest of development; thanks to the works of modern embryology. The names of Isidore Geoffroy Saint-Hilaire, Dutrochet, Serres, and of Coste, recall the authors who have best elucidated these questions.

The genital organs consist essentially of two classes, which are clearly distinct, both in the man and in the woman: those of the one preponderant and essential, are developed within the abdominal cavity, and are especially designed for the secretion of the fundamental products, ovule and sperm; the other, secondary and accessory, perform their functions outside of the abdominal cavity, in the neighborhood of the pubic arch. These two parts during intra-uterine life are independent of each other; they have a different location, their vessels and nerves arising from opposite sources, and are even separated from each other by structures, whose normal *rôle* is to disappear at a given moment of fœtal evolution. When this disappearance does not take place, and the adhesion abnormally persists, there will result some variety of these arrests of development which we have designated under the name of hermaphrodism.

Take for example the conformation of the female sexual apparatus; one of the two groups of organs is superficial and comprises the external genital organs; the other is profound, intra-pelvic, and comprises the organs of generation properly speaking, or the internal genital organs; these two groups are connected by an intermediate organ, the vagina, interposed between the *bas-fond* of the bladder anteriorly and the rectum posteriorly, in such a manner that it is a mixed organ, having a particular development, a special formation : should this conduit become obliterated, and the internal genital organs become atrophied, then the external genital parts present an exaggerated development, and there will result that variety of hermaphrodism which is usually designated under the name of apparent hermaphrodism in the female sex; because then the clitoris acquires an exaggerated size, it is grooved, as in those individuals who are the subjects of hypospadias, and the meatus, also, opens almost at the base of the clitoris.

But the results of embryology will enable us to penetrate still further in the study of these facts. In an embryo of thirty-five days, the Wolffian bodies, situated on the sides of the vertebral column, are completely developed: on their external face, there is a distorted conduit which terminates above in a wide orifice, inferiorly it opens in the bladder alongside of that of the opposite side ; it is this conduit, called the spermduct or oviduct, which, later, will constitute the *vas deferens* or *Fallopian tube*, according to the sex. On the internal surface of the same Wolffian bodies is observed a swelling which grows from day to day : this will be the testicle or ovary ; this swelling has two filamentous processes, the superior of which goes to be attached to the oviduct, the inferior goes to be inserted in the pubic spine. Later, if the individual is of the male sex, the spermduct goes to unite with the testicle, then the vas deferens exists ; on the contrary, if the person is of the female sex, the Fallopian tube remains isolated from the ovary.

Inferiorly, the oviduct and the spermduct are arranged in two different ways ; the spermduct remains separated from its fellow, and, after traversing an enlargement which will become the prostate, opens to the inferior part of the urinary passages. The prostate, which, according to some authors, (Meckel, Is. Geoffroy Saint-Hilaire,) ought especially to be regarded as the analogue of the uterus, is a structure formed by the two vasa deferentia, just as will be seen the uterus is manifestly a formation of the two oviducts. In reality, these two conduits unite to form a single cavity which will be the cavity of the uterus; should the fusion be incomplete, there will result a vice of conformation very rare, a partitioned uterus ; of which a remarkable specimen is in the museum of Orfila.

The preceding observations arise from the facts already prominently advanced by Dutrochet, in his report on the memoir of Is. Geoffroy Saint-Hilaire, viz., the complete analogy, the absolute identity which exists in the sphere of the internal generative organs during the first months of intra-uterine life, in the blastema even ; in the primitive seat, the absolute identity which exists in the internal part of the Wolffian bodies ; the analogous structure of the conduits which constitute the Fallopian tube or the vas deferens ; even the anatomical elements entering into the structure of that which will some day be the ovary or testicle, the uterus or the prostate. It is not until the eighth month that the testicles leave the places which they occupied, and during the ninth month they traverse the inguinal canal in order to reach the scrotum. This remarkable analogy is even more striking, when we come to consider the development of the external generative organs. It is at about the sixth week that there is seen to form, from the animal layer, or the external layer of the blastodermic membrane, a fissure which is common to the genito-urinary organs and the apparatus of defecation, and which has received the name of *cloaca;* this fissure will always extend to meet the *cul-de-sac* formed by the inferior extremity of the intestinal layer. From each side of this antero-posterior fissure, according to Coste, appear superiorly two projections: rudimentary corpora cavernosa; inferiorly, there are two other smaller projections, which in the man are the origin of the scrotum, and in the woman of the labia majora. These two upper processes unite at their upper surface, leaving on their inferior surface a groove for the canal of the urethra in the male, while in the female the groove remains permanent. If, in the male, the urethral canal fails to be developed beneath the corpora cavernosa, which constitute the penis, there will result a vice of conformation which we have already spoken of as hypospadias. The two inferior processes remain distinct from each other; but we may know at once, as Professor Richet* has said, "whatever the future sex may be, no difference exists; and it is not till later, in uniting, that the two processes form the scrotum in the male, while in the female, separated by the longitudinal fissure, they form the labia majora."

Later, a septum divides into two portions the fissure, which to this time was designated under the name of cloaca; the anterior division of this fissure, (uro-genital) continues to be a cavity into which the excretory canals of the genital and urinary organs open.

Finally, in the male, the union of the two lateral processes takes place, while in the female the persistent labia continue to limit an opening which now takes the name of vulva.

Thus, from this observation, we learn that, in the first moments of their existence, the external genital organs of all fœtuses have the same conformation, to that degree, even, that the clitoris in the fœtus of four months is as large as the penis of the fœtus of the same age. Two phases, in the development, are then observed: in the first, the separation of the two lateral parts, which to this time had developed towards each other, remains permanent; whilst in the second phase, and only in the male fœtus, the union of the two lateral parts is effected; in this connection, we repeat the judicious remark of Dutrochet†: "With respect to the conformation of the external genital organs, every man was at first a woman."

Should the two borders of the uro-genital fissure fail

* Richet. Traité d' Anat. Méd-Chirurg.

† Dutrochet. Acad. des Sciences.

to approach each other, there will be beneath the penis, often of diminished size, an infundibuliform cavity, bordered on each side by a fold which will resemble the labium majus much more than ordinarily obtains in these cases ; the urethra failing to effect its fusion beneath the united corpora cavernosa, there is hypospadias, and often cryptorchidia. There results then that vice of conformation which is made the subject of this study ; and which allows us still to conclude formally that, in the great majority of instances, the hermaphrodites ought to be referred to the male sex.

Thus, as Serres has remarked in his *Organogénésie*, the fœtuses of the mammifera pass by different degrees of organic formation, which correspond in their transitory phases to the normal, constant state of the creatures that are placed lowest in the organic scale ; thus is not the monstrosity often, he asks, the persistence of one of the transitory phases of the fœtal organization ?

How are these arrests of development, located in the various parts of the genital organs, to be explained ? Here again, we are enabled to accept the considerations given by Serres, regarding the preponderant influence of the vessels on the development of the organs. According to the origin of these vessels, he divides them into three orders, each one corresponding to a subdivision of the generative organs ; the generative organs themselves being constituted by the association of six principal sections. Moreover, this fact has been established by Is. Geoffroy Saint-Hilaire, and that, too, in the male sex as well as in the female. The superior division receives its arteries from the aorta, through the spermatic artery or the utero-ovarian artery (ovarian, spermatic) ; the middle division, from the hypogastric artery, through the uterine arteries, the arteries of the prostate, the internal pudic, etc.; finally, the inferior or superficial division has its own arterial network arising from the external iliac artery or the femoral, through the external pudic artery. Upon these data, however, his explanations are rather more philosophical than positive. Serres thus founds his conclusions, in order to show how the arrests of development, which constitute hermaphrodism, may be located either in the two inferior symmetrical divisions, (for example, absence of union of the two lateral folds which should form the scrotum), or the two superior divisions, (absence, or arrest of development of the ovary or testicle), accordingly as the arteries of the inferior or superior divisions are obliterated or are of diminished calibre.

In the same manner, he explains the facts which we have considered under lateral or bisexual hermaphrodism; facts that nearly all modern authors reject as being the result of insufficient observation. Serres presumes that, in these cases, the vascular irrigation being unequally supplied to these three lateral divisions, there will result upon one side an incomplete development, and upon the other, on the contrary, a true hypertrophy; consequently a formation of female organs upon one side, and male organs upon the opposite side.

(To be continued.)

